# Translation and psychometric evaluation of the Persian version of the nurses’ quality of life scale: a validation study in Iran

**DOI:** 10.1186/s12912-024-01839-7

**Published:** 2024-03-18

**Authors:** Mehrdad Yousefnezhad, Habib Shareinia, Elahe Lal Kheirkhah, Moosa Sajjadi

**Affiliations:** 1 Department of Medical Surgical Nursing, School of Nursing, Gonabad University of Medical Sciences, Gonabad, Iran; 2 Department of Gerontology, School of Nursing, Social Development and Health Promotion Research Center, Gonabad University of Medical Sciences, Gonabad, Iran; 3 Department of Nursing and midwifery, sabzevar university of medical sciences, sabzevar, Iran; 4 Department of Medical-Surgical Nursing, Faculty of Nursing, Nursing Research Center, Gonabad University of Medical Sciences, Gonabad, Iran

**Keywords:** Validation, Quality of life, Nurses, Translation, Psychometric evaluation, Iran

## Abstract

**Background:**

The quality of life for nurses can be significantly impacted by various occupational factors that Influence their working conditions and professional performance. The current study aimed to translate and validate the Persian version of the Nurses’ Quality of Life Scale.

**Material and method:**

In this cross-sectional research, the Nurses’ Quality of Life Scale (NQOLS) was utilized to assess the quality of life among 500 employed nurses in hospitals in the cities of Gonabad and Sabzevar. The translation process of the NQOLS followed the model proposed by Wild et al. The content validity of the Persian version of the scale was evaluated using the Content Validity Index (CVI) and Content Validity Ratio (CVR). Structural validity was assessed using exploratory and confirmatory factor analyses. Internal consistency reliability was assessed using Cronbach’s alpha, while test-retest reliability was determined using the Intraclass Correlation Coefficient (ICC). Data analysis was conducted using SPSS version 26 and LISREL version 8.8 software.

**Results:**

The exploratory factor analysis of the Persian version of NQOLS revealed six factors that accounted for 62.15% of the total variance. The structural validity of the extracted factors was confirmed through confirmatory factor analysis. The Cronbach’s alpha coefficient and ICC for the entire questionnaire were 0.91 and 0.95, respectively.

**Conclusion:**

The findings of the present study suggest that the Persian version of the NQOLS exhibits sufficient validity and reliability. Therefore, it can be used as an effective tool for measuring and examining the quality of life among nurses in Iran.

## Background

The human workforce is essential for the success of organizations, including healthcare organizations [1]. Nurses play a crucial role in the healthcare system by ensuring the continuity of care and promoting health. However, nursing is a high-stress profession that can significantly impact the overall health and well-being of nurses [2, 3].

The demanding nature of nursing work and the associated stressors can have a negative impact on the physical and emotional well-being of nurses. This can lead to physical and mental illnesses, as well as negative self-perceptions and attitudes. Ultimately, these factors diminish the overall quality of life for nurses [3].

Assessing the quality of life within communities is crucial for comprehending the health of societies and ensuring the well-being of individuals and communities [4]. Occupational factors and work conditions in the nursing profession can significantly impact the quality of life of nurses [5]. Quality of life, as defined by the World Health Organization, encompasses various dimensions such as physical and mental health, social relationships, and environmental well-being [6]. Nurses with a lower quality of life tend to have poorer job performance [7].

Given the influence of living conditions and stressors on nurses’ health and quality of life, it is essential to prioritize their well-being, as they play a crucial role in protecting the health of society [8]. Neglecting the mental health and quality of life of nurses can lead to decreased motivation, despair, and various physical and psychological disorders [9]. This can contribute to high job turnover rates, resulting in significant costs for the healthcare system [10].

While researching the quality of life of nurses is important, it is a complex concept with multiple dimensions influenced by factors such as age, culture, gender, education, socioeconomic status, and more [11]. Studying all these factors in working individuals poses challenges. Research studies on the quality of life of nurses raise awareness among policymakers and inform better planning for improved working conditions [12]. These studies emphasize the significance of addressing this complex issue, which is influenced by factors such as age, culture, education, and socioeconomic status [10].

Implementing a comprehensive tool to assess the quality of life of nurses in Iran would enable focused evaluation and identification of areas for improvement. Prioritizing the quality of life for nurses is crucial for enhancing job satisfaction and improving patient care [13]. Assessing the quality of life of nurses in Iran requires the use of a comprehensive tool that can effectively evaluate and identify areas for improvement. Previous studies have used various tools to evaluate the quality of life of nurses. One such tool is the Work-Related Quality of Life (WRQoL) questionnaire, which has been shown to be valid and reliable in assessing the quality of work life for nurses. Additionally, the Short Form Health Survey (SF-36) and its Iranian counterpart, the SF-12, have been extensively utilized to assess the quality of life in various populations, including nurses [14–16]. However, while these tools offer valuable insights, they may not fully capture all aspects of nurses’ quality of life. Therefore, there is still a need for a valid and specific tool that comprehensively integrates the multidimensional aspects of nurses’ quality of life [17, 18]. Therefore, there is a need for a valid and specific tool.

The Nurse Quality of Life Scale (NQOLS) is a suitable tool that integrates various dimensions of nurses’ quality of life into four domains. It takes into account the unique circumstances of nurses and provides a precise and comprehensive assessment [19]. Translating and validating the Persian version of the NQOLS in this study aims to facilitate a more precise evaluation of nurses’ quality of life in Iran. It can serve as a basis for additional research and interventions aimed at improving the quality of life for nurses and enhancing patient care.

## Methods

### Study design

The present study is cross-sectional research that carried out in the cities of Gonabad and Sabzevar in northeastern Iran from April to November 2023.

### Scale

The Nurse Quality of Life Scale (NQOLS) was developed by Sili et al. [19]. to evaluate the quality of life of nurses. The scale comprises 28 items categorized into four domains: “physical” (8 items), “emotional” (8 items), “work” (6 items), and “social” (6 items). Respondents rate their satisfaction using a 4-point Likert scale (1 = very dissatisfied, 2 = dissatisfied, 3 = satisfied, and 4 = very satisfied). The total score varied between 28 and 112. Higher scores indicate a better quality of life. The scale showed good fit and reliability, with omega coefficients ranging from 0.84 to 0.92 for components and 0.93 for the overall scale [19].

### Demographic questionnaire

The demographic questionnaire aimed to gather essential information about the background characteristics of the participants. The survey included various items related to age, gender, level of education, marital status, shift work schedule, work experience, overtime hours, willingness to work overtime, income level, and the specific department where the nurses worked. These demographic variables were considered important factors for capturing a comprehensive understanding of the sample’s characteristics and their potential influence on the research outcomes.

### Scale translation

**Fig. 1:**
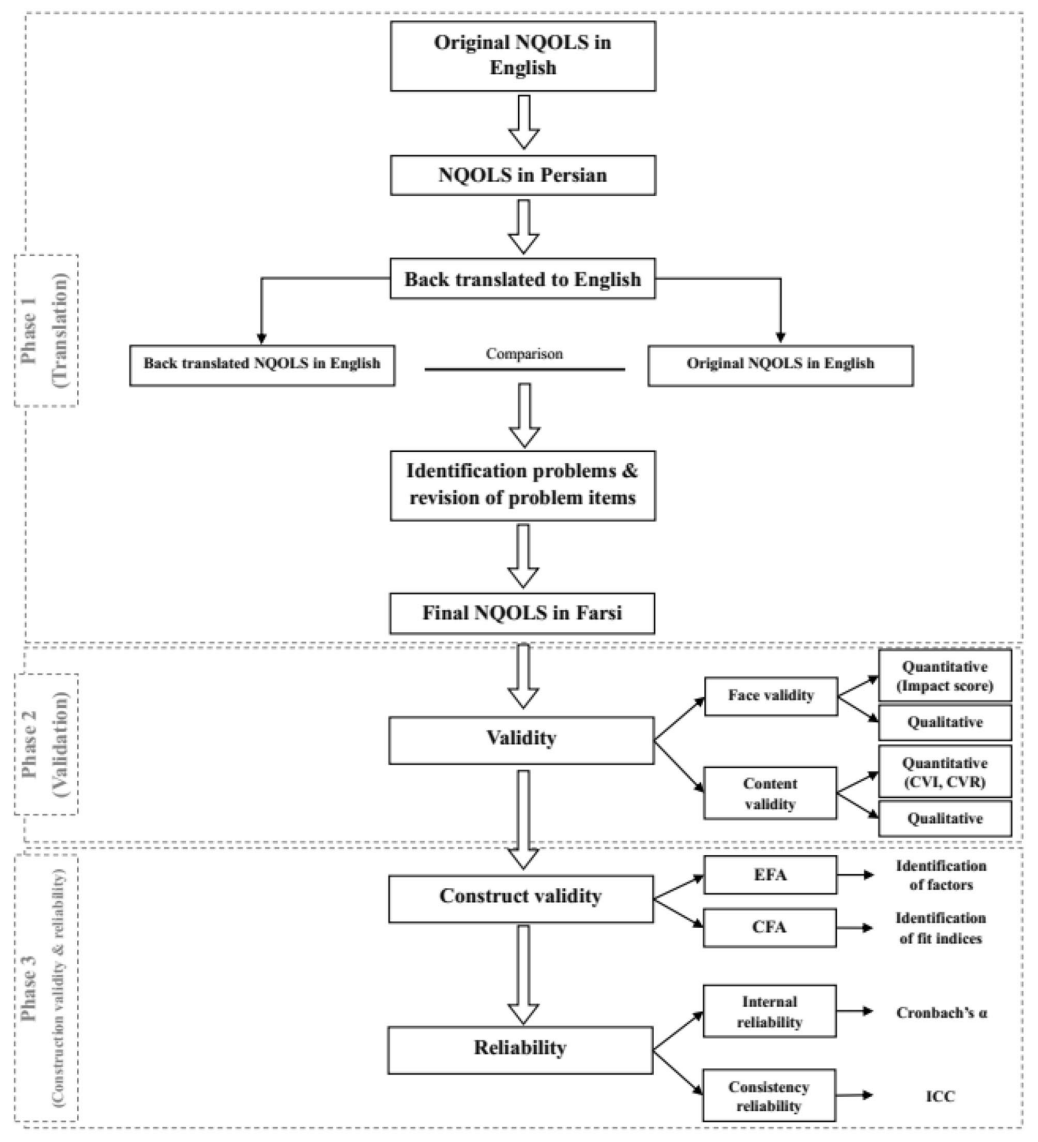
Flow chart of the psychometric assessment process of the Persian Version of the NQOLS

The scale was translated following the guidelines of Wild et al(2005) [20], and with permission from the original developers. Two translators, proficient in English and Persian, translated the scale into Persian. Their translations were compared and modified to create a final version. Two additional translators, fluent in both languages, then translated the Persian version back into English. The original tool designer then validated the translated version. The translated scale was subsequently validated for face validity, content validity, construct validity, internal consistency, and scale stability (Fig. 1).

### Validity

Face validity: The scale translated into Persian was given to 20 nurses to evaluate the face validity using a qualitative method, and the items were assessed in term of difficulty level, appropriateness level and wording ambiguity. Quantitative Face validity was obtained using the impact score method via the Likert scale. In the impact score method, an impact score was obtained by multiplying the frequency of an item by the impact of an item. An impact score above 1.5 means that the item was suitable for later analyses and will be kept [21].

Content validity: To assess the content validity using a qualitative and quantitative method. 10 experts (professional nurses and expert in the field of psychometrics) were asked to give their opinion on language accuracy, word suitability, and item arrangement. Quantitative assessment utilized the Content Validity Ratio (CVR) and Content Validity Index (CVI), with a focus on relevance [22]. A minimum CVR value of 0.62 was required. Overall, both the face and content validity were rigorously examined to ensure the accuracy and appropriateness of the research instrument.

### Participants

The study included all nurses working in different clinical departments at educational hospitals in Gonabad and Sabzevar. A sample of 500 nurses completed the Persian version of the Nurses’ Quality of Life Scale for psychometric evaluation. Participants were selected by convenience and purposive sampling based on study criteria. The inclusion criteria included having a bachelor’s degree in nursing, being employed in clinical departments, providing consent to participate, and having at least one year of clinical experience. Non-participation and incomplete questionnaires were excluded. For exploratory factor analysis, it is recommended to have a sample size of approximately 100 to 250 individuals, or 2 to 20 individuals per item [23]. For confirmatory factor analysis, it is recommended to have a sample size of 150 to 500 individuals [24]. Accordingly, 500 nurses were finally selected, who were randomly divided into two sub-samples: 200 for exploratory factor analysis and 300 for confirmatory factor analysis.

### Data analysis

Descriptive statistics were utilized to report participant characteristics. Mean and standard deviation were used to analyze quantitative variables, while frequency and percentage were used for qualitative variables.

### Construct validity

Construct validity was assessed using exploratory factor analysis (EFA) and confirmatory factor analysis (CFA). An exploratory factor analysis (EFA) was conducted on a sample of 200 individuals. The suitability of the data for exploratory factor analysis (EFA) was assessed using the Kaiser-Meyer-Olkin (KMO) measure and Bartlett’s test of sphericity. CFA was conducted on a sample of 300 individuals using LISREL software version 8.8. Model fit was assessed using several indices, including Incremental Fit Index (IFI), Normed Fit Index (NFI), Non-Normed Fit Index (NNFI or TLI), Comparative Fit Index (CFI), Goodness-of-Fit Index (GFI), chi-square to degrees of freedom ratio, and Root Mean Square Error of Approximation (RMSEA). These indices are commonly used to assess the fit of the CFA model. Two methods were employed to evaluate the reliability of the scale in this study. Internal consistency reliability and test-retest reliability. Internal consistency reliability was assessed using Cronbach’s alpha coefficient with a sample of 200 volunteer nurses. A minimum acceptable value of 0.70 was established for item acceptance. Stability and reliability were evaluated using a test-retest method with a two-week interval for 30 nurses. The intraclass correlation coefficient (ICC) was calculated to measure the reliability of stability. Values between 0.50 and 0.75 were considered to indicate good reliability, while values exceeding 0.75 were considered to indicate high reliability.

### Ethical consideration

The research proposal was approved by the Ethics Committee of Gonabad University of Medical Sciences (code: IR.GMU.REC.1402.002), and informed consent was obtained from participants. The questionnaires used in the study were designed to ensure anonymity and did not contain any personal identifying information.

## Results

### Demographic characteristics

A total of 44.4% of the participants were male. Participants who were married accounted for 61.8% of the sample; 81.4% of the participants in the academic degree program held a bachelor’s degree. A total of 47.4% of the participants were Official nurses. Other demographic characteristics of the participants are presented in Table 1 (Table 1).

**Table 1:** Demographic characteristics of the participants (*N* = 500)

Variable	Categories	N (%)
Gender	Men	222 (44.4)
	Female	278 (55.6)
City	Gonabad	220 (44)
	Sabzevar	280 (56)
Academic degree	bachelor’s degree	407 (81.4)
	Master’s degree	92 (18.2)
	PhD	1 (0.2)
Marital status	Married	309 (61.8)
	Single	191 (38.2)
Income	Low	230 (46)
	Moderate	261 (52.2)
	High	9 (1.8)
Shift status	Temporary	447 (89.4)
	Stable	53 (10.6)
Overtime	Yes	404 (80.8)
	No	96 (19.2)
Willingness to work overtime	Yes	153 (30.6)
	No	347 (69.4)
Employment status	Temporary	183 (36.6)
	Contractual	80 (16)
	Official	237 (47.4)
Ward of work	Internal	(20/2) 101
	Surgery	(14/2) 71
	Intensive care unit	(27/6) 138
	Pediatric	(8/2) 41
	Emergency department	(22) 110
	Psychiatric	(7/8) 39
**Variable**		Mean ± SD
Age		31.03 ± 6.27
Overtime (hours/month)		62.63 ± 29.90
Work experience (year)		7.35 ± 6.09

### Item analysis

The correlation coefficient between the score of each item and the total score of the translated scale was 0.403 ~ 0.619. Cronbach’s α coefficient of the translated scale was 0.911, and after deleting any item, Cronbach’s α coefficient of the translated scale ranged from 0.905 to 0.910, without any specific value. The mean (SD) item score and skewness and kurtosis values of the Chinese version of the PHBS are shown in Table 2. The skewness and kurtosis values showed that the dataset conformed to a normal distribution.

**Table 2:** Mean (SD) scores with skewness and kurtosis, item analysis for the Persian NQOLS

Item	Item score (SD)	Item-total correlation	Cronbach’s Alpha if item deleted	Skewness	Kurtosis
1	2.31 (0.71)	0.403	0.910	− 0.064	-0.394
2	2.31 (0.72)	0.413	0.910	-0.325	-0.682
3	2.54 (0.70)	0.417	0.910	-0.615	-0.085
4	2.50 (0.70)	0.467	0.909	-0.370	-0.206
5	2.57 (0.62)	0.410	0.910	-0.429	-0.038
6	2.67 (0.68)	0.463	0.909	-0.317	0.088
7	2.70 (0.67)	0.475	0.909	-0.436	0.280
8	2.41 (0.71)	0.483	0.910	-0.018	-0.254
9	2.51 (0.73)	0.465	0.909	-0.522	-0.068
10	2.49 (0.74)	0.498	0.908	-0.511	-0.154
11	2.56 (0.68)	0.524	0.908	-0.523	-0.004
12	2.51 (0.75)	0.648	0.905	-0.215	-0.296
13	2.63 (0.62)	0.528	0.908	-0.748	0.398
14	2.52 (0.69)	0.619	0.906	-0.300	-0.174
15	2.62 (0.71)	0.529	0.908	-0.020	-0.264
16	2.74 (0.67)	0.591	0.907	-0.307	0.169
17	2.70 (0.70)	0.526	0.908	-0.564	0.358
18	2.6 (0.67)	0.467	0.909	-0.401	0.024
19	2.52 (0.75)	0.522	0.908	-0.297	-0.284
20	2.52 (0.70)	0.479	0.909	-0.160	-0.198
21	2.65 (0.69)	0.480	0.908	-0.415	0.120
22	2.61 (0.73)	0.476	0.909	-0.572	0.080
23	2.87 (0.59)	0.549	0.908	-0.693	0.667
24	2.84 (0.62)	0.561	0.907	-0.369	0.549
25	2.77 (0.70)	0.470	0.909	-0.348	0.161
26	2.89 (0.64)	0.527	0.908	-0.580	1.101
27	2.79 (0.65)	0.501	0.908	-0.951	0.448
28	2.13 (0.79)	0.480	0.909	0.049	-0.841

### Construct validity (EFA, CFA)

Face validity was qualitatively assessed with input from nurses, while content validity was evaluated with input from experts, nursing stakeholders, and psychologists. Minor modifications were made to the wording of items without changing their meaning. Content validity was also quantitatively assessed using CVI and CVR values, which were obtained from the opinions of ten experts (Table 3). In quantitative face validity the impact score was obtained from 2.96 to 4.32, that means all items were suitable. No items were excluded due to these validity measures.

**Table 3:** CVR, CVI and factor loading of the Persian NQOLS

Item	Item content	Content validity		Factor loading					
		CVR	CVI	F1	F2	F3	F4	F5	F6
11	I am satisfied with my resistance in stressful situations	1	1	0.621					
12	I am satisfied with my mood	1	1	0.665					
13	I am satisfied with my mental efficiency	1	1	0.631					
14	I am satisfied with my emotional stability	0.8	1	0.583					
15	I am satisfied with my self-confidence	1	1	0.761					
16	I am satisfied with my ability to solve problems	1	1	0.757					
17	I am satisfied with my psychological independence	0.8	1	0.688					
18	I am satisfied with my capacity to control myself	0.8	1	0.638					
				E.V: 15.27%					
23	I am satisfied with my relationships with others	1	1		0.711				
24	I am satisfied with my role in my family	1	1		0.674				
25	I am satisfied with the relationships I have with my relatives	1	1		0.851				
26	I am satisfied with the relationships I have with my friends	1	1		0.697				
27	I am satisfied with the relationships I have with my colleagues	1	1		0.628				
					E.V: 10.99%				
19	I am satisfied with my type of job	1	1			0.767			
20	I am satisfied with the way my work is organized	1	1			0.737			
21	I am satisfied with my professional role	1	1			0.820			
22	I am satisfied with what I do in my job	1	1			0.804			
28	I am satisfied with my financial situation	1	1			0.328			
						E.V: 10.64%			
1	I am satisfied with the amount of my sleep	1	1				0.801		
2	I am satisfied with the quality of my sleep	1	1				0.838		
3	I am satisfied with the quality of my nutrition	1	1				0.648		
4	I am satisfied with my eating habits	1	1				0.588		
							E.V: 9.77%		
5	I am satisfied with my physical health	1	1					0.495	
6	I am satisfied with my physical appearance and physical image	1	1					0.665	
7	I am satisfied with my physical skills	0.8	1					0.711	
8	I am satisfied with my level of physical activity	1	1					0.741	
								E.V: 8.33%	
9	I am satisfied with the number of my sexual relations	1	1						0.858
10	I am satisfied with the quality of my sexual relations	1	1						0.844
									E.V: 7.13%

E.V: Explained variance

**Fig. 2:**
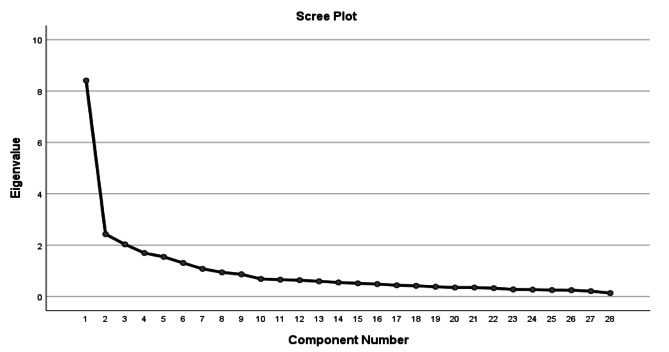
Scree plot for the Persian version of the Persian Version of the “Nurses’ Quality of Life Scale”

In the exploratory factor analysis (EFA) with a sample size of *N* = 200, the Kaiser-Meyer-Olkin (KMO) measure was 0.866, indicating sufficient sample adequacy [25]. The Bartlett’s test of sphericity was significant (χ^2^ = 2629.789, df = 378, *p* < 0.05), indicating that the data is suitable for factor analysis. Six factors with eigenvalues > 1 were identified, explaining 62.15% of the total variance of the Nursing Quality of Life Scale (NQOLS). The six-factor structure, labeled as Emotional (F_1_), Social (F_2_), Career (F_3_), Self-care (F_4_), Physical (F_5_), and Sexual (F_6_) dimensions, was confirmed (Table 3; Fig. 2).

**Fig. 3:**
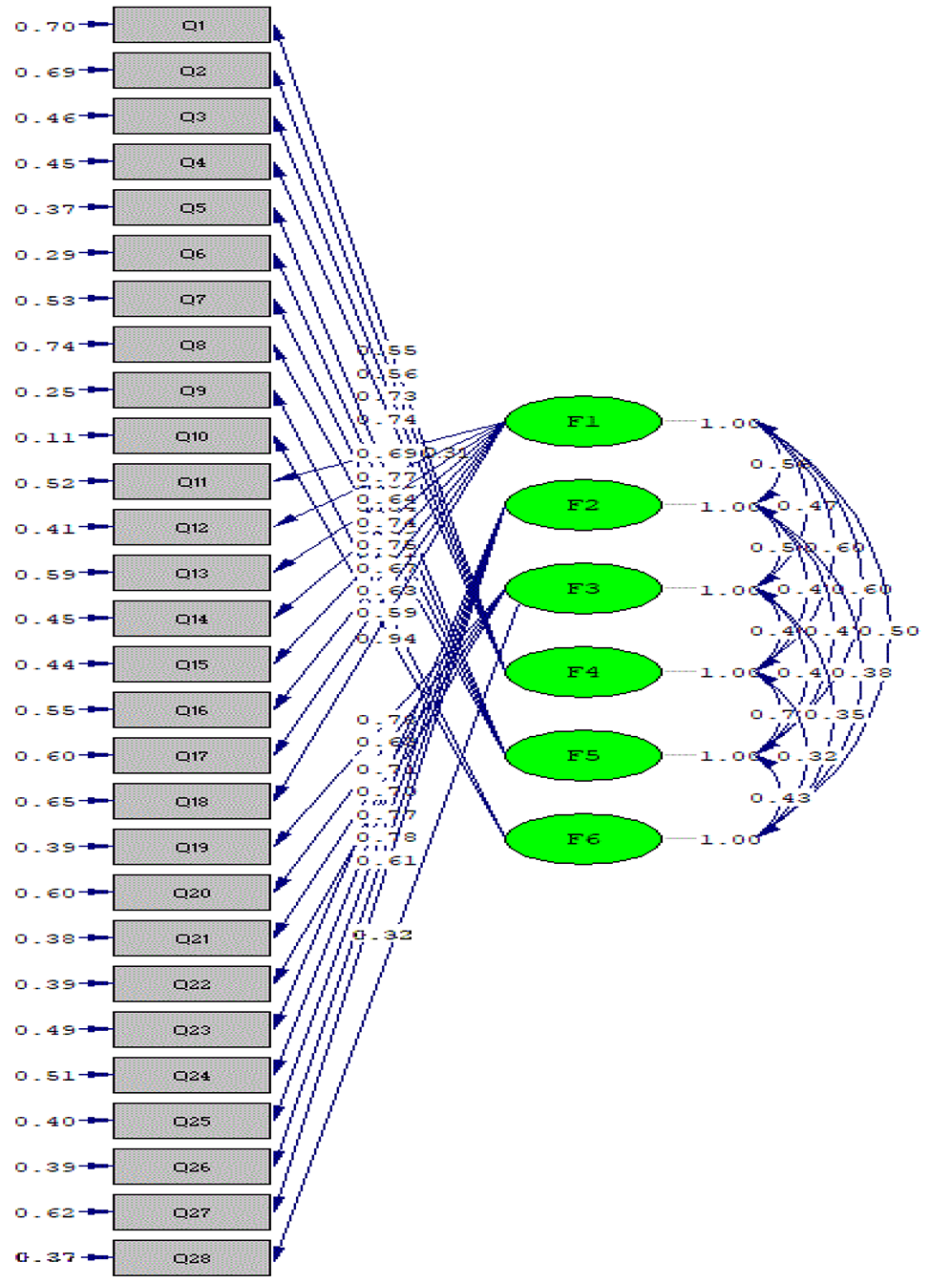
Results of confirmatory factor analysis in standard mode (*n* = 300)

To evaluate the appropriateness of the model derived from the EFA with a six-factor structure, a CFA was performed. The model fit indices for the CFA are presented in Table 3. Based on the CFA results, it was determined that the obtained model demonstrated a good fit according to the fit indices, confirming the six-factor structure for the Persian version of the NQOLS (Table 4; Fig. 3).

**Table 4:** Model fit indices of CFA for the Persian version of the NQOLS

Index	IFI	GFI	CFI	NFI	RMSEA	χ^2^/df (*p* value)
value	0.96	0.84	0.96	0.93	0.068	783.38/335 (< 0.0001)

IFI: Incremental Fit Index, GFI: Goodness of Fit Index, CFI: Comparative Fit Index,

NFI: Normed Fit Index, RMSEA: Root Mean Square Error of Approximation

The Cronbach’s alpha values obtained for the scale factors ranged from 0.764 to 0.911, indicating satisfactory internal consistency for the Persian version of the NQOLS. The minimum threshold of 0.764 was met for each factor, indicating sufficient reliability. The overall scale exhibited a Cronbach’s alpha value of 0.911, indicating strong internal consistency for the entire scale. To evaluate the test-retest reliability, we calculated the intraclass correlation coefficient (ICC), which yielded a value of 0.958 for the overall scale. This indicates a high level of stability in repeated measurements. (CI: 95%, 0.912–0.980, *p*-value < 0.001) (Table 5).

**Table 5:** ICC and Cronbach α of the Persian NQOLS

Factors	Number of items	Cronbach α	ICC- (CI:95%)
Emotional	8	0.875	0.965 - (0.927–0.984)
Social	5	0.837	0.936 - (0.855–0.970)
Career	5	0.826	0.976 - (0.949–0.988)
Self-caring	4	0.764	0.938 - (0.865–0.972)
Career	5	0.826	0.976 - (0.949–0.988)
Sexual	2	0.910	0.942 - (0.878–0.972)
Total scale	28	0.911	0.958 - (0.912–0.980)

*ICC: intraclass coefficient correlation*

## Discussion

The study aimed to examine the psychometric properties and factor structure of the translated Nurses’ Quality of Life Scale among Iranian nurses. The findings indicate that the Persian version of the scale exhibited suitable validity and reliability within the Iranian nursing community. However, no previous research has specifically focused on adapting the scale to the cultural context of Iranian nurses. Furthermore, there is limited research on the psychometric properties of the scale in other countries and languages beyond the original Italian study.

Quality of life, now recognized as an important indicator of health, refers to the factors that contribute to a satisfying life [26]. It is a dynamic concept that evolves over time [27]. Quality of life is defined as the combination of characteristics that are valuable to an individual and contribute to a sense of comfort, well-being, and satisfaction. These characteristics support the development and maintenance of physical, emotional, and cognitive functioning. It enables individuals to maintain their abilities in meaningful life activities [28]. Nursing, by its very nature, presents unique challenges and issues for practitioners, which can significantly impact their quality of life [29].

The present study’s findings on the face and content validity determination indicate that all items of the scale have been confirmed to be relevant, clear, and simple after undergoing minor modifications. Furthermore, the obtained CVI values indicate that the Persian version of the NQOLS possesses a highly suitable content validity, demonstrating its ability to measure the quality of life among Iranian nurses effectively.

The original NQOLS version, designed and validated by Sili et al. in Italy, comprises 28 items and 4 factors [19]. However, in our study, after conducting exploratory factor analysis and thoroughly examining the results, the questionnaire with 28 items (without excluding any items from the original questionnaire) and 6 factors was deemed more appropriate than other alternatives. The findings from the exploratory factor analysis revealed that the six factors of the nurses’ quality of life scale collectively account for 62.15% of the total variance. Regarding the selection of factor names, careful consideration was given to the content of the respective items. The factor names were chosen in line with the original version of the scale and with reference to the nursing foundations of Koozar and Erb [30], aiming to select the most suitable names.

Based on the objective of translating and validating measurement tools, the aim is to ensure their cultural appropriateness within a specific society. Therefore, in the current study, in line with the cultural background of Iranian nurses, a six-factor structure was derived for this instrument. It should be noted that validation studies of quality of life measurement tools in different languages may not necessarily yield the same number of items, factors, or item placement as the original versions [14, 31]. In our study, some items were allocated to different factors compared to the original version of the questionnaire. This discrepancy could be attributed to cultural or linguistic variations.

The fit indices for the six-factor structure of the Nurses’ Quality of Life Scale, including NFI, CFI, GFI, IFI, RMSEA, X^2^/df, and *p*-value (< 0.0001), demonstrated an acceptable fit [32] for the extracted six-factor model in the present study. In accordance with the findings of our study, Sili et al. (2022) have validated the four-factor structure of the Nurses’ Quality of Life Scale among Italian nurses in the community using confirmatory factor analysis [19].

In the present study, the reliability of the tool was assessed using two methods: internal consistency (Cronbach’s alpha coefficient) and stability (test-retest). The Cronbach’s alpha coefficients ranged from 0.74 to 0.91 for the subscales and 0.91 for the overall scale. Furthermore, the ICC for the entire tool was higher than 0.8 suggest that the scale has a high internal consistency [33]. Thus, based on the study results, it can be concluded that the Persian version of the Nurses’ Quality of Life Scale exhibits high reliability for implementation in the Iranian nursing community. Sili et al. (2022) utilized the omega reliability coefficient to examine the internal consistency reliability of the NQOLS. Consistent with our findings, their study yielded omega reliability coefficients ranging from 0.84 to 0.92 for the factors and 0.93 for the overall scale, indicating the scale’s reliability in measuring nurses’ quality of life [19].

Additionally, the psychometric analysis conducted in this study suggests that the Persian version of the Nurses’ Quality of Life Scale possesses strong validity for utilization within the Iranian nursing community. The scale aims to encompass various dimensions of quality of life, including physical, emotional, work-related, sexual, social, and family aspects. The scale items and factors were developed in consideration of the unique and specific circumstances encountered by nurses. In the design of the NQOLS, efforts were made to employ a minimal number of items with concise wording in order to facilitate ease of administration and save time [19].

### Limitations

There were several limitations in this study that need to be noted. Firstly, the sample size was limited to nurses from only two cities within one province, which may limit the generalizability of the results to the entire country. Secondly, the data collected relied on self-reporting through the NQOLS scale, which introduces the potential for participants to inaccurately answer the questions. Lastly, due to the novelty of the scale, the researcher faced challenges in finding sufficient resources and studies in this specific field for a more comprehensive discussion.

## Conclusion

The findings of current study showed that the Persian version of the Nurses’ Quality of Life Scale has excellent levels of validity and reliability among the Iranian nursing community. Furthermore, the results of face and content validity assessments indicate that this tool possesses a visually appealing appearance and is suitable for evaluating the quality of life among Persian-speaking nurses. Considering its favorable psychometric properties, ease of administration, and ability to specifically assess the quality of life in nurses, it is recommended to utilize this scale in various clinical and research settings, including both clinical practice and research projects.

To further strengthen the validity of the Persian version of the Nurses’ Quality of Life Scale, we suggest conducting additional studies to validate the scale in other provinces across Iran. This will help ensure the generalizability of the scale’s validity across different regions within the country.

## Data Availability

The datasets used and/or analyzed during the current study are available from the corresponding author on reasonable request.

## Abbreviations

NQOLS: Nurses’ Quality of Life Scale
EFA: Exploratory Factor Analysis
CFA: Confirmatory Factor Analysis
NFI: Normed Fit Index
IFI: Incremental Fit Index
CFI: Comparative fit index
GFI: goodness of fit index
RMSEA: root-mean-square error of approximation
CVI: content validity index
CVR: content validity ratio
ICC: Interclass Correlation Coefficient
χ^2^: Chi square
Df: degree of freedom
